# The effect of the Sport Education Model in physical education on student learning attitude: a systematic review

**DOI:** 10.1186/s12889-024-18243-0

**Published:** 2024-04-02

**Authors:** Junlong Zhang, Wensheng Xiao, Kim Geok Soh, Gege Yao, Mohd Ashraff Bin Mohd Anuar, Xiaorong Bai, Lixia Bao

**Affiliations:** 1 Department of Sports Studies, Faculty of Education Studies, University Putra Malaysia, 43400 Seri Kembangan, Malaysia; 2 School of Physical Education, Huzhou University, Huzhou, 313000 China; 3 The 19, Middle School, Haigang District, Qinhuangdao, 066000 China; 4 Department of Professional Development and Continuing Education, Faculty of Education Studies, University Putra Malaysia, 43400, Seri Kembangan, Malaysia

**Keywords:** Sports education curriculum, Learning attitude, Cognitive, Affective

## Abstract

**Background:**

Evidence indicates that the Sport Education Model (SEM) has demonstrated effectiveness in enhancing students' athletic capabilities and fostering their enthusiasm for sports. Nevertheless, there remains a dearth of comprehensive reviews examining the impact of the SEM on students' attitudes toward physical education learning.

**Purpose:**

The purpose of this review is to elucidate the influence of the SEM on students' attitudes toward physical education learning.

**Methods:**

Employing the preferred reporting items of the Systematic Review and Meta-analysis (PRISMA) statement guidelines, a systematic search of PubMed, SCOPUS, EBSCOhost (SPORTDiscus and CINAHL Plus), and Web of Science databases was conducted in mid-January 2023. A set of keywords associated with the SEM, attitudes toward physical education learning, and students were employed to identify relevant studies. Out of 477 studies, only 13 articles fulfilled all the eligibility criteria and were consequently incorporated into this systematic review. The validated checklist of Downs and Black (1998) was employed for the assessment, and the included studies achieved quality scores ranging from 11 to 13. The ROBINS-I tool was utilized to evaluate the risk of bias in the literature, whereby only one paper exhibited a moderate risk of bias, while the remainder were deemed to have a high risk.

**Results:**

The findings unveiled significant disparities in cognitive aspects (*n* = 8) and affective components (*n* = 12) between the SEM intervention and the Traditional Teaching (TT) comparison. Existing evidence suggests that the majority of scholars concur that the SEM yields significantly superior effects in terms of students' affective and cognitive aspects compared to the TT.

**Conclusions:**

Nonetheless, several issues persist, including a lack of data regarding junior high school students and gender differences, insufficient frequency of weekly interventions, inadequate control of inter-group atmosphere disparities resulting from the same teaching setting, lack of reasonable testing, model fidelity check and consideration for regulating variables, of course, learning content, and unsuitable tools for measuring learning attitudes. In contrast, the SEM proves more effective than the TT in enhancing students' attitudes toward physical learning.

**Systematic review registration:**

(https://inplasy.com/) (INPLASY2022100040).

## Introduction

In recent years, the "student-centered" teaching model, as a more effective alternative to the traditional "teacher-centered" teaching model, has gained increasing attention and recognition from education scholars and departments worldwide [1, 2]. Metzler [3] identified a series of "student-centered" teaching models based on constructivism and social learning theories, each developed for specific course objectives [4, 5]. Furthermore, it is widely acknowledged that instructional models are in a constant state of development, involving the generation, testing, refinement, and further testing processes under different educational objectives. These instructional models are designed to enable students to acquire a depth and breadth of knowledge in physical education [6]. In this regard, a series of instructional models have been identified as effective means to achieve specific objectives. Consequently, numerous studies have established that placing students at the center of the instructional process is the most effective approach [7], allowing for the assessment of the impact of these models on students' learning in physical education. For instance, Cooperative Learning (CL), rooted in the idea of learning together with others, through others, and for others [8], aims to promote five essential elements [9]: interpersonal skills, processing, positive interdependence, promoting interaction, and individual responsibility. The underlying concept of Teaching Game for Understanding (TGFU) involves shifting the focus from technical aspects of gameplay to the context (tactical considerations) through modification of representation and exaggeration [4, 10]. Emphasizing placing learners in game situations where tactics, decision-making, and problem-solving are non-negotiable features, despite incorporating skill practice to correct habits or reinforce skills [11], TGFU is structured around six steps: game, game appreciation, tactical awareness, decision-making, skill execution, and performance. Teaching for Personal and Social Responsibility (TPSR), designed by Hellison [12], aims to cultivate personal and social responsibility in young people through sports activities, defining four major themes: integration, transfer, empowerment, and teacher-student relationships. It revolves around five responsibility goals: respecting the rights and feelings of others, effort (self-motivation), self-direction, caring (helping), and transferring beyond the "gym" [13]. The SEM comprises six key structural features: season, affiliation, formal competition, culminating events, record-keeping, and festivity. SEM seeks to provide students with authentic, educationally meaningful sporting experiences within the school sports context, aiming to achieve the goal of developing capable, cultured, and enthusiastic individuals [14]. This suggests a subtle intersection between SEM's developmental goals and enhancing students' learning attitudes (cognitive and emotional), laying the foundation for the selection of teaching model types in this study.

In previous SEM-centered reviews, the focus primarily centered on the model's positive impact on students' personal and social skills [15, 16], motor and cognitive development [16], motivation [17, 18], basic needs [18], prosocial attitudes [18], and learning outcomes [19], and it is concluded that the implementation of SEM has a positive effect on improving students' performance in these aspects. While these reviews contribute valuable insights, they exhibit certain limitations, such as a lack of comprehensive exploration of the model's impact on the cognitive and emotional dimensions in the context of school-based physical education. Therefore, our study attempts to bridge this gap by delving into the nuanced intersection between SEM and students' learning attitudes, aiming to provide a more comprehensive understanding of its impact on educational environments.

In the field of education, a focus on practical application and scholarly discourse is crucial and commendable [20, 21]. From a practical perspective, research should offer valuable resources for curriculum designers, educators, and policymakers [22–25]. In theoretical terms, the contribution of research lies in addressing gaps in the literature by elucidating dimensions within physical education that remain insufficiently explored [26]. Our study is dedicated to significantly impacting physical education teaching through the practical application and scholarly discourse surrounding SEM. By revealing the subtle interactions between SEM and attitudes, we aim to provide valuable curriculum implementation recommendations for designers, practitioners, and policymakers, filling the gaps in how SEM shapes learning attitudes in educational environments.

In the realm of attitude research, scholars have traditionally classified attitude components into three types: single-component, two-component, and three-component. Advocates of the single-component view contend that attitudes are confined to the emotional dimension. For example, Fazio and Zanna [27] define attitude as "an evaluative feeling caused by a given object" (p. 162). Two-component researchers posit that attitudes comprise cognition and emotion, with the affective component measuring emotional attraction or feelings toward the object, and the cognitive component representing beliefs about the object's characteristics [28, 29]. Bagozzi and Burnkrant [30] compared the effectiveness of one-component and two-component attitude models, concluding that incorporating both cognitive and emotional dimensions enhances attitude effectiveness. On the contrary, proponents of the three-component perspective argue that attitudes encompass cognition, emotion, and behavior, suggesting that cognitive and emotional responses to an object influence behavior. However, the three-component view has faced skepticism, with some researchers finding that attitude measurement explains only about 10% of behavior variance. Studies reporting higher correlations often focus on attitudes and behavioral intent rather than explicit behavior itself [31–33]. Our research places a deliberate emphasis on investigating the intersection between the SEM and attitudes to address a noticeable gap in the existing scholarly landscape. While none of the reviewed literature approached the subject from an attitude theory perspective, we prioritize this theoretical framework, acknowledging that attitudes significantly influence student learning [16, 34]. Consequently, the exploration of the interplay between SEM and attitudes is considered indispensable for attaining a thorough comprehension of SEM's potential impact in educational contexts. By integrating attitude theory into this inquiry, there is an aspiration to unveil nuanced insights into the cognitive and emotional dimensions influenced by SEM, thereby enriching the understanding of the model's pedagogical implications.

## Methods

The chosen systematic review approach in this study aims to enhance the reader's understanding of the research methodology, thereby strengthening the overall scientific rigor of the study [35].

### Protocol and registration

This review adheres to the guidelines set forth by the Preferred Reporting Project for Systematic Review and Meta-Analysis (PRISMA). The review has been registered on the International Registry Platform for Systematic Review and Meta-Analysis Programmes (INPLASY) under the registration number INPLASY2022100040. More information about the review can be found at the following link: https://inplasy.com/.

### Search strategy

In October 2004, Siedentop initiated SEM workshops, attracting widespread attention from scholars both domestically and internationally, marking the beginning of SEM practices [36, 37]. Subsequently, in many advanced countries such as the United States, New Zealand, Australia, and the United Kingdom, SE has become a mainstream approach in physical education instruction [38]. Therefore, the retrieval period for this review is set from October 2004 to December 2023, encompassing relevant articles published during this timeframe. A systematic search of four electronic databases was conducted for relevant articles: SCOPUS, PubMed, EBSCOhost (SPORT Discus and CINAHL Plus), and Web of Science. The search aimed to identify studies on the effects of SEM on attitudes toward physical education learning. We employed advanced search methods and added the following search terms: ("Sport Education Model" OR "Sport Education" OR "Sport season") AND ("learning attitude" OR "sports attitude" OR "cognitive" OR "cognition" OR "usefulness" OR "importance" OR "perceptions" OR "affective" OR "emotional" OR "enjoyment" OR "happiness" OR "well-being" OR "Blessedness" OR "subjective well-being") AND ("student" OR "pupil" OR "scholastic" OR "adolescent" OR "teenager"). The search expressions were combined using logical operators. We also sought assistance from librarians in the field to ensure comprehensive results. Furthermore, we manually examined the reference lists of the included studies to identify additional relevant literature and validate the effectiveness of our search strategy.

### Eligibility criteria

We employed the Picos framework, encompassing Population, Intervention, Comparison, Outcomes, and Study Design, as the inclusion criteria for this systematic review (Table 1). Furthermore, the selected literature adhered to the following additional criteria: (i) it comprised full English texts published in peer-reviewed journals; (ii) the interventions were conducted within the context of physical education, with a comprehensive description of the intervention process and content; (iii) the effects of the SEM and TT on students' learning attitudes (cognitive and emotional) were compared on at least one dimension; (iv) quasi-experimental designs employing objective tests and measurements, along with studies presenting evaluation results, were considered. Exclusion criteria encompassed studies that combined physical education models with other teaching methods or models (hybrid or invasive). Initially, the search strategy was guided by a librarian, and duplications were eliminated by importing the retrieved literature into Mendeley reference management software. Subsequently, decisions regarding literature exclusion and retention were made through the screening of titles and abstracts. Ultimately, articles deemed highly relevant were read in full. The primary outcome aimed to assess attitudes (cognitive and affective) toward physical learning based on the SEM.

**Table 1:** Inclusion criteria according to the PICOS conditions

Items	Detailed inclusion criteria
Population	students (male/female)
Intervention	Sports Education Model
Comparison	Traditional Teaching (Teacher-centered teaching model: Direct Instruction, Latent Growth Model, Traditional Style, Skill-Drill-Game, and Traditional instruction)
Outcome	Physical education learning attitude (Cognitive, affective)
Study designs	Quantitative, Both qualitative and quantitative, Experimental and Quasi-experimental, non-randomized controlled trial

**Fig. 1:**
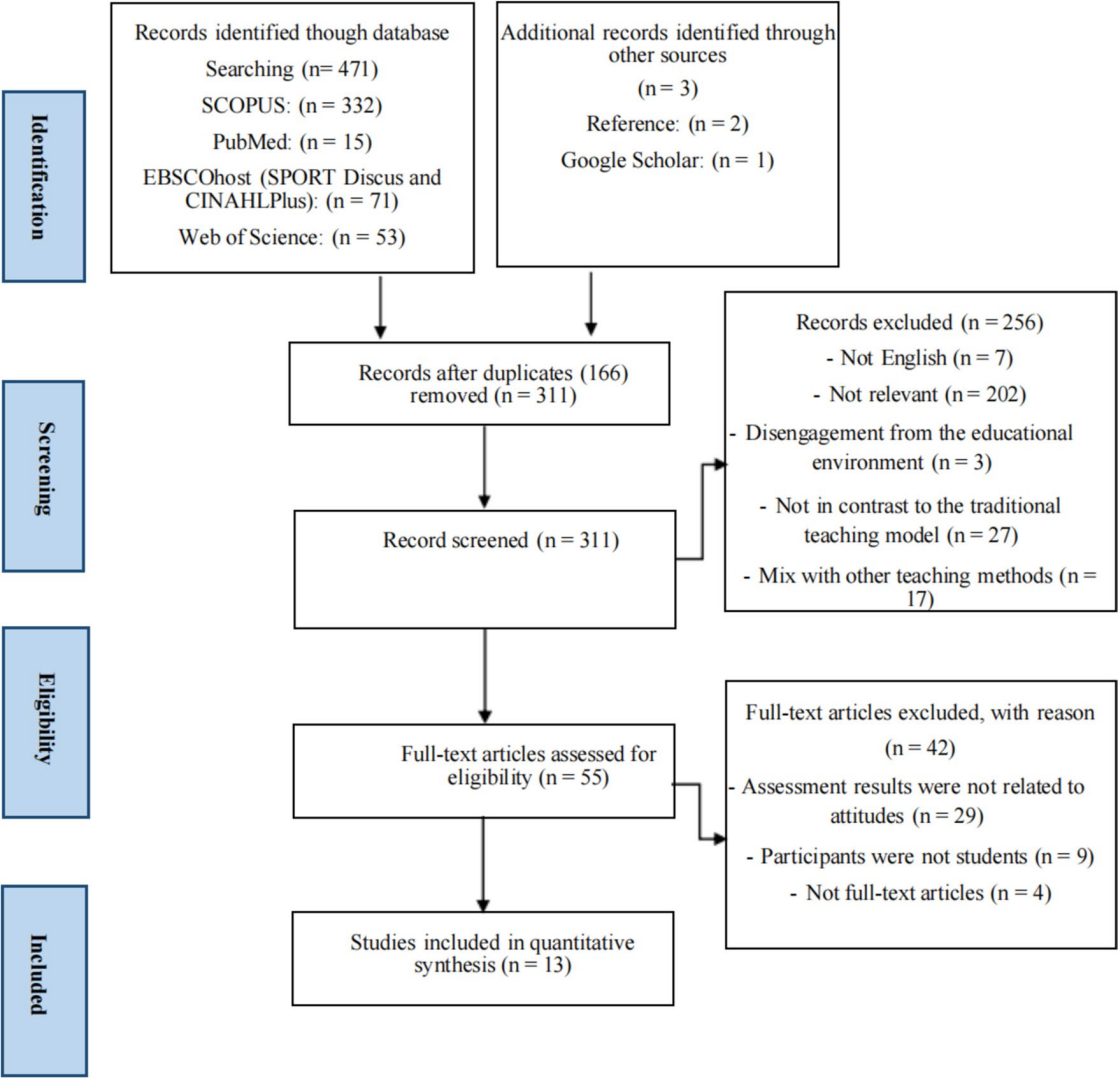
PRISMA summary of the study selection process

The search strategy was guided by a librarian, and the obtained literature was imported into Mendeley reference management software for duplicate removal. Decisions regarding literature inclusion and exclusion were made based on the screening of titles and abstracts. Articles that were deemed highly relevant were read in their entirety. The primary focus of this review was to assess attitudes (cognitive and affective) toward physical learning, specifically based on the SEM. The designation "not relevant" is employed to characterize articles subjected to thorough scrutiny, which fail to make substantive contributions to the fundamental focus of our research. More precisely, those articles deemed irrelevant were those that omitted consideration of the pivotal variables under examination, namely, cognitive and emotional dimensions. Furthermore, they were not situated within the milieu of a scholastic educational framework for physical education (SEM). This methodological approach has been instituted to uphold the establishment of a centralized and cohesive dataset requisite for subsequent analytical procedures [39] (See Fig. 1).

### Study selection

Prior to conducting the search, consultation with an experienced librarian was sought to develop an effective retrieval strategy. Following this, two independent reviewers conducted the literature search. All retrieved studies were imported into Mendeley literature management software to identify and eliminate duplicates. Initially, the literature was screened based on the titles by two independent evaluators, who excluded irrelevant studies. Subsequently, the abstracts of the initially selected literature were reviewed against pre-established inclusion criteria to determine their eligibility for inclusion in the study. Finally, the full text of the included literature was reviewed by two authors, who extracted relevant information. In the case of any disagreements, a third author (K.G.S.) was involved in the review process.

### Data extraction and quality assessment

The data extraction process involved collecting the following information: (1) author and year of publication; (2) research design, including the type of experiment or teaching project; (3) population details, such as student category, total number of students, age range, and gender distribution, as well as group size; (4) intervention characteristics, including the total number of interventions, weekly frequency of interventions, duration of each intervention, and consistency of intervention location; (5) a comparison group, typically involving the TT and country information; (6) results, which encompassed the measurement tools used, specific indicators measured, and the research findings. The collected data were independently summarized and reviewed by two authors, with the involvement of a third author to resolve any discrepancies or disagreements.

The methodological quality of the selected articles in this systematic review was assessed using the validated checklist developed by Downs and Black [40]. The checklist consisted of 27 items, which were categorized into three domains: reporting (items 1–10), validity (external validity: items 11–13; internal validity: items 14–26), and statistical power (item 27). Each item was scored, resulting in a total score ranging from 0 to 27, with higher scores indicating higher methodological quality.

In this review, the cross-sectional and longitudinal surveys were scored in detail using the Downs and Black checklist to evaluate the strengths and weaknesses of each study [40]. The scoring process involved two primary assessors independently assessing the selected studies. In case of any ambiguity or disagreement, a resolution was reached through reconciliation. If disagreements persisted, the assessment was conducted by one of the co-authors until a consensus was reached.

The classification criteria for the scores were as follows: studies with a score below 11 were considered to have low methodological quality, scores ranging from 11 to 19 indicated medium quality, and scores higher than 20 indicated high methodological quality [41]. Upon assessment, it was found that all selected articles in this review fell within the medium-quality range (see Table 2).

**Table 2:** Summary of methodological quality assessment scores

Author(s)/Year	Q1	Q2	Q3	Q4	Q5	Q6	Q7	Q8	Q9	Q10	Q11	Q12	Q13	Q14	Q15	Q16	Q17	Q18	Q19	Q20	Q21	Q22	Q23	Q24	Q25	Q26	Q27	Quality score
Wallhead & Ntoumanis (2004) [42]	1	0	1	1	0	1	1	0	0	1	UTD	UTD	UTD	0	0	0	1	1	1	1	1	0	0	0	0	UTD	0	11
Spittle & Byrne (2009) [43]	1	0	1	1	0	1	1	0	0	1	UTD	UTD	UTD	0	0	0	1	1	1	1	1	1	0	0	0	UTD	0	12
Perlman (2010) [44]	1	0	1	1	0	1	1	0	0	1	UTD	UTD	UTD	0	0	0	1	1	1	1	1	1	1	0	0	UTD	0	13
Menickelli & Hastie (2014) [45]	1	0	1	1	0	1	1	0	0	1	UTD	UTD	UTD	0	0	0	1	1	1	1	1	1	0	0	0	UTD	0	12
Wallhead et al. (2014) [23]	1	0	1	1	0	1	1	0	0	1	UTD	UTD	UTD	0	0	0	1	1	1	1	1	1	0	0	0	UTD	0	12
Cuevas et al. (2016) [46]	1	0	1	1	0	1	1	0	0	1	UTD	UTD	UTD	0	0	0	1	1	1	1	1	1	1	0	0	UTD	0	13
Fernández-Río et al. (2017) [47]	1	0	1	1	0	1	1	0	0	1	UTD	UTD	UTD	0	0	0	1	1	1	1	1	1	0	0	0	UTD	0	12
Xu et al. (2019) [48]	1	1	1	1	0	1	1	0	0	1	UTD	UTD	UTD	UTD	UTD	0	1	1	1	1	1	1	0	0	0	UTD	0	13
Zhang & Su, (2020) [49]	1	0	1	1	0	1	1	0	0	1	UTD	UTD	UTD	0	0	0	1	1	1	1	1	1	0	0	0	UTD	0	12
Viciana et al. (2020) [50]	1	0	1	1	0	1	1	0	0	1	UTD	UTD	UTD	0	0	0	1	1	1	1	1	1	1	0	0	UTD	0	13
Luna et al. (2020) [51]	1	0	1	1	0	1	1	0	0	1	UTD	UTD	UTD	0	0	0	1	1	1	1	1	1	1	0	0	UTD	0	13
Chu et al. (2022) [52]	1	0	1	1	0	1	1	0	0	1	UTD	UTD	UTD	0	0	0	1	1	1	1	1	1	0	0	0	UTD	0	12
Iserbyt et al. (2023) [53]	1	1	1	1	0	1	1	0	0	1	UTD	UTD	UTD	0	0	0	1	1	1	1	1	1	0	0	0	UTD	0	13

Evaluation checklist: http://dx.doi.org/10.1136/jech.52.6.377

### The studies risk of bias

The Risk of Bias in Non-randomized Studies-of Interventions (ROBINS-I) tool encompasses seven evaluation areas, which are further divided into three distinct stages: pre-intervention, intervention, and post-intervention. The pre-intervention stage includes two evaluation areas: confounding bias and selection bias of participants. The intervention stage focuses on the evaluation of bias in the classification of interventions. The post-intervention stage comprises four evaluation areas: bias due to deviations from intended interventions, bias due to missing data, bias in the measurement of outcomes, and bias in the selection of reported results. Each evaluation area is composed of multiple signaling questions, amounting to a total of 34 signaling questions.

## Results

### Methodical quality

The articles underwent assessment using the validated checklist developed by Downs and Black (1998): 11–13 (mean = 12.38; median = 12; mode = 12 & 13). All the articles demonstrated a medium level of quality, indicating their suitability for inclusion in this review. Furthermore, it suggests the potential for higher-quality articles in future studies. Among the thirteen included articles, five were published within the last three years, constituting one-third of the included literature. This observation highlights the ongoing research interest and significance of the SEM in the investigation of various teaching models. In terms of the Hypothesis/aim/objective, participant characteristics, interventions, main findings, data variability, probability values, statistical tests, detailed intervention descriptions, reliable outcome measures, participant source (*n* = 12), participant grouping (*n* = 11), and random allocation (*n* = 3) were adequately addressed. However, aspects such as reporting measurement outcomes in the introduction or methods section, confounder distribution, adverse events following the intervention, characterization of lost-to-follow-up patients, data analysis, blinding of participants and assessors, adjustment for confounding, and identification of chance results with a probability less than 5% (*n* = 0) were not thoroughly addressed. Although the implementation of blind subjects, therapists, and assessors in teaching experiments poses challenges, future research should strive for higher quality and stronger levels of evidence [23].

After a detailed reading of the literature that meets the inclusion criteria of this review and the extraction and sorting of important information, it is presented in Table 3.

**Table 3:** Characteristics of the studies examined in the present review

Study	Design	Participants	Interventions	Type of teaching	Measuring instruments and main outcomes
Wallhead & Ntoumanis (2004) [42]	Pre-post Test, basketball	High school N = 51, Age:14.3 ± 0.48 yr EG = 25 (M\F) CG = 26 (M\F)	SEM F: once a week 8 classes (60 min), Same site	TT (TS) (England)	Intrinsic Motivation Inventory (IMI) SEM > TT: Affective: SEM ⬆, TT ↔ ; Cognitive: SEM ⬆, TT ↔
Spittle & Byrne (2009) [43]	Pre-post Test, Hockey, soccer	Junior high N = 115, M/F, 97/ 18 Age: 13–14 yr EG = 41 (M\F) CG = 74 (M\F)	SEM 20 classes (45 min), F: Once a week Same site	TT (DI) (Australia)	Intrinsic Motivation Inventory (IMI) SEM > TT: Affective: SEM ⬆, TT ⬇; Cognitive: SEM ⬆, TT ⬇
Perlman (2010) [44]	Pre-post Test, basketball	High school N = 78 EG = 40 (M\F) CG = 38 (M\F)	SEM 20 classes (60 min), F: Three or four times a week, Same site	TT (SDG) (United States)	Intrinsic Motivation Inventory (IMI) SEM > TT Affective: SEM ⬆, TT ↔
Menickelli & Hastie (2014) [45]	Pre-post Test, Disc Lacrosse	High school N = 40, M/F, 30/ 10 Age:15.9 ± 1.1 yr EG = 20 (M\F) CG = 20 (M\F)	SEM 20 classes F: Five times a week Same site	TT (SDG) (United States)	Intrinsic Motivation Inventory (IMI) SEM > TT (Affective and Cognitive)
Wallhead et al (2014) [23]	Pre-post Test, Ball course	High school N = 568 M/F, 310/ 258 Age:14.74 ± 0.48 yr EG = 281 (M\F) CG = 287 (M\F)	SEM F: Two or three times a week 25 classes (90 min), Two schools	TT (MM) (United States)	A motivation subscale of the Academic Motivation Scale (AMS) SEM > TT: Affective: SEM ⬆, TT ↔ ; Cognitive: SEM ⬆, TT ↔
Cuevas et al. (2016) [46]	Pre-post Test, Volleyball	High school N = 86, M/F, 49/ 37 Age: 15.65 ± 0.78 yr EG = 43 (M\F) CG = 43 (M\F)	SEM 19 classes F: Twice a week Same site	TT (DI) (Spain)	Intention to be Physically Active Scale (IPAS) SEM > TT: Affective: Positive affect: SEM ↔ , TT ↔ ; negative affect: SEM ↔ , TT ↔
Fernández-Río et al. (2017) [47]	Pre-post Test, Ultimate-Frisbee	High school N = 217, Age: 12–17 yr M/F, 113/ 104	SEM 12 classes (55 min), F: Twice a week, Same site	TT (DI) (Spain)	Three items were developed by Duda, Fox, Biddle, and Armstrong (1992) to measure boredom Intrinsic Motivation Inventory (IMI) SEM > TT: Affective: Positive affect: SEM ⬆, TT ↔ ; negative affect: SEM ⬇, TT ↔
Xu et al. (2019) [48]	Pre-post Test, Table tennis	High school N = 64, Age: 16–17 yr EG = 36(M\F) CG = 28 (M\F)	SEM 16 classes F: Once a week Same site	TT (China)	The attitude questionnaire SEM > TT: Cognitive: SEM ⬆, TT ↔ ; Affective: SEM ⬆, TT ↔
Zhang & Su, (2020) [49]	Pre-post Test, Body movements	College N = 60, M/F, 20/40 Age: 20.52 ± 0.8 yr EG = 30(M\F) CG = 30 (M\F)	SEM F: Once a week 10 classes (55 min), Same site	TT (LGM) (China)	Physical activity enjoyment scale (PACES) SEM > TT (Affective)
Viciana et al. (2020) [50]	Pre-post Test, Volleyball	High school N = 123, M/F, 60/ 63 Age: 14–15 yr 3EG (M\F) 2CG (M\F)	SEM 12 classes (50 min), Two different schools F: Not mentioned	TT (DI) (Morocco)	The Spanish version of the Sport Satisfaction Instrument (SVSSI); SEM > TT: Affective: (negative affect) SEM ⬇, TT ↔ ; Cognitive: SEM ⬆, TT ↔
Luna et al. (2020) [51]	Pre-post Test, Polskie Ringo (a game)	primary school N = 146, M/F, 67/ 79 Age: 10.78 ± 1.07 yr EG = 87 (M\F) CG = 59 (M\F)	SEM 18classes (50 min) F: Not mentioned Same site	TT (DI) (Spain)	Positive and Negative Affect Scale (PANASN) SEM > TT Affective: Positive affect: SEM ⬆, TT ⬆; negative affect: SEM ⬇, TT ↔
Chu et al. (2022) [52]	Pre-post Test, basketball	College N = 60, M/F, 28/ 32 EG = 30 (M\F) CG = 30 (M\F)	SEM 16classes (45 min), F: Twice a week, Same site	TT (China)	The ARCS Learning Motivation Scale The Physical Education Affection Scale (PEAS) SEM > TT Affective: SEM ⬆, TT ↔ , Cognitive: SEM ⬆, TT ⬆ (SEM = TT)
Iserbyt et al. (2023) [53]	Pre-post Test, basketball	College N = 85, M/F, 70/ 15 EG = 2 classes(M\F) CG = 2 classes(M\F)	SEM 16classes (60 min), Same site	TT (Belgium)	Cognitive: SEM ⬆, TT ↔ The ALT-PE data were collected using momentary time sampling for each team by trained coders Cognitive: SEM ⬆, TT ↔

> : The overall teaching effect has a significant advantage; ⬆: The results of pre—and post-test in the group were significantly improved; ↔ : There was no significant change in the test results before and after the group; ⬇: There was no significant decrease in the test results before and after the group

*N* number of participants in the experiment, *M*/*F* male and female, *EG* experimental group, *CG* control group, *TT* Traditional Teaching Model, *DI* Direct Instruction, *LGM* Latent Growth Model, *TS* Traditional Style, *SDG* Skill-Drill-Game, *TI* Traditional Instruction, *MM* multiactivity model, *F* Weekly intervention frequency, *ALT-PE* Academic Learning Time Physical Education

### The studies risk of bias

The bias risk assessment results are summarized in Table 4, which includes information such as author/date, field of study, study type, risk assessment tool, and overall rating. The main sources of bias identified were confounding factors and outcomes measurement. The evaluation revealed that only two experimental studies in the Confounders field had a moderate risk of bias, while the rest had a high risk of bias. All included literature demonstrated low risk in terms of subject selection, classification of recommended interventions, and deviation from established interventions. Furthermore, one-third of the literature showed low-risk missing data [23, 42, 50, 51], while other studies did not provide relevant information. Lastly, nearly a third of the literature showed missing data for low-risk.

**Table 4:** Study risk of bias

**Author(s)/Date**	**I**	**II**	**III**	**IV**	**V**	**VI**	**VII**	**L**	**M**	**S**	**C**	**NI**	**Rating**^a^	**Research area**
Wallhead & Ntoumanis (2004) [42]	Moderate	Low	Low	Low	Low	Serious	Moderate	4	2	2	0	0	Serious	Affective & cognitive
Spittle & Byrne (2009) [43]	Serious	Low	Low	Low	NI	Serious	Moderate	3	1	2	0	1	Serious	Affective & cognitive
Perlman (2010) [44]	Serious	Low	Low	Low	NI	Serious	Moderate	3	1	2	0	1	Serious	Affective
Menickelli & Hastie (2014) [45]	Serious	Low	Low	Low	NI	Serious	Moderate	3	1	2	0	0	Serious	Affective & cognitive
Wallhead et al. (2014) [23]	Serious	Low	Low	Low	Low	Serious	Moderate	4	1	2	0	0	Serious	Affective & cognitive
Cuevas et al. (2016) [46]	Serious	Low	Low	Low	NI	Moderate	Moderate	3	2	1	0	1	Serious	Affective
Fernández-Río et al. (2017) [47]	Serious	Low	Low	Low	NI	Serious	Moderate	3	1	2	0	1	Serious	Affective
Xu et al. (2019) [48]	Serious	Low	Low	Low	NI	Serious	Moderate	3	1	2	0	1	Serious	Affective & cognitive
Zhang & Su, (2020) [49]	Serious	Low	Low	Low	NI	Serious	Moderate	3	1	2	0	1	Serious	Affective
Viciana et al. (2020) [50]	Moderate	Low	Low	Low	Low	Moderate	Moderate	4	3	0	0	0	Moderate	Affective & cognitive
Luna et al. (2020) [51]	Serious	Low	Low	Low	Low	Serious	Moderate	4	2	1	0	0	Serious	Affective
Chu et al. (2022) [52]	Serious	Low	Low	Low	NI	Serious	Moderate	3	1	2	0	1	Serious	Affective & cognitive
Iserbyt et al. (2023) [53]	Serious	Low	Low	Low	NI	Serious	Moderate	3	1	2	0	1	Serious	Cognitive

ROBINS-I tools (Sterne et al., 2016) checklist items for non-randomized controlled studies related to bias in the following domains: I. Confounders; II. Participants selection; III. Classification of interventions; IV. Deviations from intervention; V. Missing data; VI. Outcomes measurement; VII. Results selection

*L* Low risk, *M* Moderate risk, *S* Serious risk, *C* Critical risk, *NI* No information

^a^Rating: Declaring a study to be at a particular level of risk of bias for an individual domain will mean that the study as a whole has a risk of bias at least this severe

### Overview of sports and experiment design

All thirteen papers included in this review utilized a pre-posttest design. The sports covered in these studies encompassed basketball, volleyball, soccer, ultimate Frisbee, table tennis, hockey, Polskie ringo, ball games, and body movements. Some studies examined two exercise programs [23, 43], while the majority of research focused on basketball [44, 52, 53]. The participants in the course experiments were primarily college and high school students, with a limited number of studies investigating primary and junior high school students. The distribution of participants included college students (3), high school students (8), primary school students (1), and junior high school students (1). The sample sizes in these studies ranged from 40 to 508. Since the selected studies were teaching experiments, most of them involved mixed-sex classes, with four studies not specifying the gender of the students. Only one study established three experimental classes and two control classes [50], while the remaining studies had one experimental class and one control class. The number of interventions ranged from 8 to 25, with each intervention lasting between 45 and 90 min.

The majority of studies in the selected literature directly applied the SEM as the intervention. Five of the studies incorporated constructivism theory [48], self-determination theory [23, 44, 47], and ARCS learning motivation theory [52]. None of the literature investigated from the perspective of attitude theory. Furthermore, none of the selected studies mentioned the teaching standards or syllabus used to design the course content, nor did they provide explanations for the rationale behind the experimental teaching content. The number of interventions in the trials ranged from 8 to 25, with up to half of the studies using fewer than 18 interventions [42, 47–50, 52, 53], the recommended class hours for large unit teaching are not met [54]. The duration of each intervention was most commonly reported as 45 or 60 min [42–44, 47, 49–53]. The frequency of weekly interventions varied from 1 to 5, but the majority of studies implemented interventions once a week [23, 42, 43, 46–49]. The intervention frequency was generally low, and there was a scarcity of studies with higher intervention frequency. With the exception of one article that conducted the intervention in two schools without providing an explanation [50], the remaining studies were conducted within the same school.

The control classes in the selected literature implemented similar TT and forms, despite variations in naming used by scholars from different countries or even within the same country. The TT employed in the control classes were mainly Direct Instruction in Australia [43, 46, 47, 51, 52], Morocco [50], and Spain [42–44], In China, the traditional teaching models were referred to as TT [48, 52] and Latent Growth Model [49]; Traditional Style in the United States and England [42], American Skill-drill-game [44, 45], and multiactivity model [23].

### Measuring instruments and main outcomes

The findings of this investigation were classified based on the impact of the SEM on various aspects of students' attitudes toward physical education: cognitive and affective domains. Through the segregation of subjects and constituents from prior research, the favorable and unfavorable indicators of affective and cognitive dimensions were predominantly derived from the existing body of literature.

### The effect of SEM on student cognitive

In this literature review, it was evident that all the included studies reached a unanimous conclusion that the overall effectiveness of the SEM surpassed that of the TT. Among these studies, eight of them specifically evaluated students' cognitive performance [23, 42, 43, 45, 48, 50, 52]. Various assessment instruments were employed, such as the Intrinsic Motivation Inventory (IMI) [42, 43, 45], the Amotivation subscale of the Academic Motivation Scale (AMS) [23], the attitude questionnaire [48], the Spanish version of the Sport Satisfaction Instrument (SVSSI) [50], the ARCS Learning Motivation Scale, the Physical Education Affection Scale (PEAS) [52], and the ALT-PE data were collected using momentary time sampling for each team by trained coders [53].

The study participants encompassed junior high school students [43], high school students [23, 42, 45, 48, 50] and College students [52, 53]. Most of these investigations revealed that following the intervention of the physical education course, the cognitive abilities of students in the intervention group exhibited significant improvement, surpassing those of the control group instructed through the TT. Conversely, no significant changes were observed within the control group before and after the experiment [23, 42, 48, 50]. Nevertheless, one study reported a significant decrease in cognitive abilities among students in the control group before and after the experiment [54], the other two studies showed that both the experimental and control groups showed significant improvements, but the experimental group showed significantly greater improvements [52, 53].

### The effect of SEM on student's affective

In this comprehensive review, all the included studies examined students' affective aspects. The assessment instruments employed were as follows: Intrinsic Motivation Inventory (IMI) [42–45, 47], Amotivation subscale of the Academic Motivation Scale (AMS) [23], Intention to be Physically Active Scale (IPAS) [46], the attitude questionnaire [48], Physical activity enjoyment scale (PACES) [49], the Spanish version of the Sport Satisfaction Instrument (SVSSI) [50], Positive and Negative Affect Scale (PANASN) [51] and the Physical Education Affection Scale (PEAS) [52].

The study participants encompassed primary school students [51], Junior high school students [43], high school [23, 42, 44–48, 50, 51] and College students [49, 52]. Out of the 12 studies, four reported positive and/or negative interests or enjoyment among students. Among these, two studies indicated that the experimental group students exhibited significantly higher positive affect than the control group students [47, 51]. However, the measurement results varied within the control group. One study reported no significant improvement [47], while another study showed significant improvement, but the effect was significantly greater in the experimental group compared to the control group [51]. Furthermore, one study demonstrated no significant difference between the two groups as the test indicators did not exhibit significant changes before and after the experiment [46].

Regarding the investigation of negative affect, three studies reported that the experimental group students exhibited significantly lower negative affect compared to the control group [47, 51], with a significant decrease in negative affect observed in the experimental group while no significant change was noted in the control group. Additionally, one study showed no significant difference and no significant improvement in the test results between the two groups before and after the experiment [46].

Among the remaining eight studies, it was not specified whether the investigation focused on positive or negative effects. Among them, two studies solely compared the improvement effects between the experimental and control groups without conducting intra-group comparisons before and after the experiment, and the results revealed that the experimental group exhibited significantly better outcomes than the control group [45, 49]; the remaining six studies conducted comparisons not only between groups before and after the experiment but also within each group. Five studies demonstrated a significant increase in the affected index of the experimental group, while the control group exhibited no significant change [23, 42, 44, 48, 52], and one study revealed that the experimental group displayed a significant improvement, while the control group experienced a significant decline [43].

## Discussion

This paper presents a comprehensive review of the effects of the SEM on students' attitudes towards physical education. Its aim is to distinguish this study from other published research on the application of the SEM interventions among students. The findings indicate that the SE model has the potential to enhance students' attitudes toward physical education in terms of cognition and affect. However, certain factors such as the lack of data on junior high school students and gender differences, the frequency and duration of intervention per week, the variation in the learning environment across groups taught in the same setting, the rationale behind the course content, and the selection of tools for measuring learning attitudes may influence the experimental outcomes. Nonetheless, considering the positive results observed in these studies, is SEM an effective way to interfere with students' attitudes toward physical education learning? In conjunction with the information presented in the "Results" section, this review offers a detailed analysis of the impact of various dimensions of student attitudes toward physical education learning.

### Overview of sports and experiment design

As anticipated, eleven out of the thirteen studies included in this review focused on ball games, which aligns with the competitive nature of these sports [55]. This choice is well-suited to the seasonal characteristics of the Sports Education Model (SEM) [56, 57]. When considering gender comparisons, incorporating gender research can enhance the reliability of experimental findings [58, 59]. However, in all the studies included, the majority of researchers only used mixed experimental and control groups, without comparing gender distinctions. If significant differences exist in the effect of SEM on the learning attitudes of students of different genders, it would significantly impact the accuracy of the experimental results.

Regarding the frequency, number, and duration of each intervention, some scholars have suggested that these factors may have different effects on the experimental outcomes [60], However, among the thirteen studies reviewed, the largest number of interventions was only 25 [23], and most studies had fewer than 20 interventions. Most studies had fewer than 18 interventions. This deviates from the use of large unit teaching advocated by some scholars to enhance students' systematic cognition and learning experience of a sports event [54, 61]. In the reform of the school curriculum, the State Council of China issued the Curriculum Standards for Physical Education and Health for Compulsory Education (2022 edition) for students, which also clearly mentioned that the length of class hours for large units should not be less than 18 lessons.

In terms of the rationality of classroom teaching form and content, Hastie et al. [62] developed an Instructional Checklist to evaluate the effectiveness of the SEM and TT. However, only four of the included studies addressed this aspect [46, 47, 50]. Regarding the selection of measurement tools, none of the studies examined students' learning attitudes using scales developed based on attitude theory. According to the two-component proponents of attitude, attitude theory defines attitude as the affective and cognitive (positive or negative) evaluation of individuals toward the object of attitude [28–30, 63]. Failing to assess student attitudes using survey instruments developed based on the structural composition of attitudes is problematic, as these instruments may not accurately measure attitudes [64]. The critical concern regarding the assessment of student attitudes using survey instruments developed based on the structural composition of attitudes requires a more thorough explanation. This is particularly important because relying on instruments that do not align with the multi-dimensional nature of attitudes, encompassing affective, cognitive, and conative components, may lead to inaccurate measurements [64]. To elaborate further, historical quantitative investigations in physical education pedagogy often utilized instruments such as Kenyon's [65] or Simon and Smoll's [66], which might not capture the complete construct of attitude. For instance, Kenyon's instrument conceptualizes physical activity rather than attitude as a multidimensional construct, while Simon and Smoll's instrument, developed for adults, may not be entirely valid for children. This unidimensional perspective on attitude, focusing solely on the affective dimension, is problematic, as it overlooks the multi-component nature of attitude, as acknowledged in studies by Gonzàles [67], Mohsin [68], and Oppenheim [69]. Therefore, future research endeavors should delve into the intricacies of attitude assessment tools, considering the developmental differences and the multidimensional nature of attitudes to ensure comprehensive and accurate measurement in the context of physical education pedagogy.

### The effect of SEM on student cognitive

The existing literature provides sufficient evidence to support the significant superiority of physical education courses over TT in enhancing students' cognition of physical education learning. The cognitive dimension refers to individuals' evaluation of concepts and beliefs related to specific people, things, and objects, forming a multi-perspective system [32, 49]. The development of ideas and beliefs relies on a solid foundation of knowledge about people and things. Students' cognition of physical education learning serves as a prerequisite for fostering positive attitudes toward physical education [70]. However, among the eight studies included in this review that examined the cognitive components of attitudes, seven studies concluded that SEM and TT had a more significant impact on improving students' perception of attitudes toward physical education learning [23, 42, 43, 45, 48, 50, 53]. Most of these studies indicated that students' perception of physical education learning did not change significantly under TT. Only one study found that both SEM and TT showed significant improvements before and after the experiment, with no significant difference in the degree of improvement between them [52]. However, it is noteworthy that the study by Chu et al. [49] lacked a thorough examination of the model fidelity for both the SEM and TT. The absence of a robust fidelity check raises concerns about the reliability and validity of the observed improvements reported in both SEM and TT groups before and after the experiment. Without ensuring that the implemented instructional models were faithfully executed as intended, it becomes challenging to attribute the observed improvements solely to the effectiveness of the instructional methods. Consequently, the study reports significant improvements in both SEM and TT without a discernible difference in the degree of improvement between them. This underscores the importance of conducting comprehensive model fidelity checks to enhance the credibility and interpretability of research findings, particularly when comparing the effectiveness of different instructional models in educational settings. Although most studies support the significant superiority of the SEM in enhancing students' perception of physical education learning compared to traditional instruction, it is important to note that five out of seven studies were conducted with high school students, limiting the generalizability of the findings to broader populations. This represents a crucial gap in the existing literature regarding learning cognition in physical education. Furthermore, despite having mixed-gender classes, the studies did not include a comparative analysis of students from different genders. Therefore, it is necessary to conduct additional comparative studies on the SEM and TT, encompassing various learning stages and considering the cognition of physical education learning among students of different genders, to enrich the breadth of results.

### The effect of SEM on student's affective

The majority of sports scholars hold the view that the SEM is superior to the TT in fostering students' emotional experiences in sports learning. The affective dimension pertains to the emotions and emotional experiences of individuals based on cognitive factors related to specific people, things, or objects, such as interest or enjoyment [32, 49]. By comparing SEM and TT, eleven out of the thirteen studies analyzing improvements in student physical education learning confirmed that SEM significantly outperformed TT in enhancing student interest or enjoyment [23, 42–45, 47–52]. Only one study found that both SEM and TT did not lead to significant improvements in student interest or enjoyment, as there were no significant changes in test results before and after the learning social work experiment in both groups [46]. Notably, three of the studies involved opposite outcomes of positive and negative effects [46, 47, 51], and one study exclusively reported negative affect [50]. These divergent results underscore the complexity of the relationship between instructional models and students' attitudes towards physical education. Future research endeavors should delve deeper into the factors contributing to such variations, exploring potential moderating variables, instructional nuances, or contextual influences that may elucidate the observed disparities. These findings not only deserve attention for their immediate implications but also emphasize the need for nuanced investigations that can inform the refinement and optimization of instructional approaches in the field of physical education.

Moreover, among the four studies involving 20 or more interventions, three studies conducted within-group comparisons of SEM and TT before and after the experiment [23, 43, 45], and the frequency of weekly interventions varied. One study with a low intervention frequency found a significant decrease in emotional aspects among students in the TT group before and after the experiment [43]. However, two studies with high intervention frequency found no significant changes in the emotional aspects of students in the TT group before and after the experiment [23, 44]. These results contradict Chen's argument (2019) that prolonged treatment may lead to adverse emotions such as anxiety and depression. However, these limited findings do not provide strong evidence and require further validation in future studies with larger sample sizes.

## Limitations

In summary, this review presents substantial evidence supporting the superiority of the SEM over TT in enhancing students' attitudes toward physical education learning. However, there are several limitations to consider. Firstly, none of the included studies reported gender differences, which limits the richness and specificity of the research findings. Gender differences, if present, could potentially impact the accuracy of the overall results. Secondly, the studies did not address the influence of class size on teaching experiment outcomes. Determining the optimal number of students per group and the ideal number of groups is an important consideration for achieving optimal teaching effects. Inappropriate, insufficient, or excessive sample sizes can affect the quality and accuracy of experiments [71]. Thirdly, most studies did not account for the experimental environment or control participants' physical activities outside the experimental setting, which may influence students' attitudes toward physical education learning. Additionally, the studies generally did not consider the impact of factors such as climate and time on students' attitudes during the teaching experiments. Lastly, none of the studies included in this review conducted any short-term or long-term follow-up of students after the trial, making it challenging to determine the long-term effects of SEM on students' attitudes toward physical education learning.

## Conclusion

The systematic review conducted provides compelling evidence supporting the positive impact of the SEM on students' attitudes toward physical education learning. However, it is important to note that most of the literature included in this review focused on high school and college students, while there were fewer findings for other school age groups. Urgently needed are comprehensive research initiatives that prioritize investigating the impact of the SEM on attitudes towards physical education learning across diverse age groups, including primary and middle school students. This will contribute to a more inclusive understanding of SEM's effectiveness, ensuring that its benefits are explored and validated across various educational stages, thus providing a solid foundation for evidence-based instructional practices in physical education. Additionally, although SEM is an established teaching model, recent research has shown an increase in its popularity in physical education, with five out of the thirteen studies published in the last three years. Nevertheless, it is crucial to approach the results with caution due to the limitations identified in this study.

To further deepen our understanding of the effectiveness of SEM in improving students' attitudes toward physical education learning, it is imperative to address the issue of model fidelity checks for both SEM and TT. The study highlighted the absence of a thorough examination of the model fidelity in certain investigations, which raises concerns about the reliability and validity of the observed improvements reported in both SEM and TT groups before and after the experiment. Future research should prioritize rigorous fidelity checks to enhance the credibility and interpretability of research findings when comparing the effectiveness of different instructional models.

Moreover, the identified divergent outcomes in some studies, including those with opposite positive and negative effects, as well as studies reporting exclusively negative affect, underscore the complexity of the relationship between instructional models and students' attitudes towards physical education. Therefore, future investigations should explore potential moderating variables, instructional nuances, or contextual influences contributing to such variations. This comprehensive approach will not only help refine our understanding of SEM's impact on attitudes but also aid in the selection of teaching models that align with the demands of contemporary times.

To optimize the study of SEM's influence on students' physical education learning attitudes, it is recommended to increase the number and frequency of interventions appropriately. Additionally, future research endeavors should consider demographic factors such as the gender and age of the students, contributing to a more nuanced understanding of SEM's impact across different populations. This continued exploration will not only verify the advantages of SEM in promoting students' physical education learning but also enrich the research outcomes concerning the influence of SEM on students' attitudes, addressing the identified gaps and fostering advancements in physical education pedagogy.

## Data Availability

The data set supporting the conclusions of this article is included within the article.
